# Modelling the distribution of *Oxytenanthera abyssinica (A. Richard) under changing* climate: implications for future dryland ecosystem restoration

**DOI:** 10.1016/j.heliyon.2022.e10393

**Published:** 2022-08-24

**Authors:** Weldemariam Ch. Elias, Dejene W. Sintayehu, Bobasa F. Arbo, Abraha K. Hadera

**Affiliations:** a Department of Geo-Information Science, Haramaya University, Dire Dawa, Ethiopia; b College of Agriculture and Environmental Sciences, Dire Dawa, Ethiopia; c Department of Environmental Sciences, Haramaya University, Dire Dawa, Ethiopia; d Gondar Agricultural Research Center, Ethiopia

**Keywords:** *Oxytenanthera abyssinica*, Climate change, SDM (Ensemble approach), Africa

## Abstract

Bamboo is the world's most widely exploited plant resource, with significant socio-economic and cultural values. In most parts of Africa, the population is in jeopardy due to the high pressure from human and natural forces. Of these, *Oxytenanthera abyssinica* (A. Richard) is among the threatened bamboo species. Furthermore, the effect of climate change on the distribution of bamboo has not yet been adequately studied. Therefore, this study aims to model and map the current and future distribution of *O. abyssinica* in Africa under four representative concentration pathways (RCPs), such as RCP2.6, RCP4.5, RCP6, and RCP8.5. The future projections were done for the years 2050 and 2070 using SDM ensemble approaches. To model the current and future distribution of *O. abyssinica* in Africa, 737 presence data were collected from various sources. For this study, a total of eight (8) temperature and precipitation-related variables were used as inputs to the Species Distribution Model (SDM). Finally, the model performance was assessed based on the area under the curve (AUC) and true skills statistics (TSS) measures of statistics. Our results showed an upsurge in the distribution of *O. abyssinica* across the study area for the low and moderate suitability classes for the climatic conditions considered in this study. However, a steady shrinkage in the habitat was found for the higher suitability classes. The model indicated climatic-related factors such as precipitation during the cold and warm quarters (57.8%), followed by mean temperature during the coldest quarter, isothermality (41.9%) and topographic factors such as elevation and slope (31.6%) were identified as the main limiting factors for the growth of *O. abyssinica*. Precipitation and temperature during the dry period, on the other hand, had the least impact on the growth of *O. abyssinica*. Except for RCP2.6, the majority of south-western African countries and the Sahel region remain the most climatically stable ecosystems for *O. abyssinica* growth under the three climatic scenarios RCP45, RCP6 and RCP8.5. Our results revealed a steady increase in the future suitable habitat for *O. abyssinica* all over the continent under the considered climatic scenarios. Therefore, to support the future restoration of dryland ecosystems, countries should scheme a restoration policy that allows the sustainable utilization of *O. abyssinica* tree species. The future policy direction for biodiversity conservation and management should encourage the use of *O. abyssinica* as a major plant species for improving the livelihoods of people living in dryland areas.

## 1 Introduction

Bamboo is one of the world's most widely exploited natural resources, providing major benefits from different socio-economic and cultural values (Embaye, 2003a, Embaye, 2003b; INBAR, 2010; Hogarth and Belcher, 2013; Gebramlak et al., 2016). It has a potential role in providing rural and urban populations with jobs and economic development opportunities (Kassahun et al., 2003; UNIDO, 2009; Kalanzi et al., 2018). In addition to rural income and opportunities for jobs, bamboo makes a major contribution to environmental services and conservation of the environment (Embaye, 2003a, Embaye, 2003b; UNIDO, 2009; Hogarth and Belcher, 2013; Darcha and Birhane, 2015; Kalanzi et al., 2018). Bamboo forests are distinguished by a complicated network of rhizome-root systems that make them excel in keeping soil particles together effectively, preventing soil erosion and promoting water percolation (Sileshi and Nath, 2017). By intercepting rainfall and sheltering the soil from wind erosion and sun drying, the aboveground section of a bamboo forest helps mitigate erosion.

Bamboo species are native to different African countries, ranging from the east to the west. There are around 1642 to 1662 bamboo species in the world (Canavan et al., 2016; Vorontsova et al., 2016), covering more than 14 million hectares of land, and of these, 115 bamboo species are reported from 48 African countries (Bahru, 2021), covering more than 1.45 million hectares of land (Zhao et al., 2016). For example, Ethiopia accounts for 67 percent of the continent's largest African bamboo forest, which is estimated to be 7 percent of the world's total. *Oxytenanthera abyssinica* (A. Richard) and Arundinaria alpine (K. Schum.) are two bamboo species endemic to Africa (Embaye, 2003a, Embaye, 2003b). Bamboo is important for the restoration of the dryland ecosystem and socio-economic development (Mishra et al., 2014). The plant is also commonly used for house building, for soil conservation and fertility maintenance, as a source of animal feed, for human consumption, for cash revenue, and for the treatment of several diseases (Hogarth and Belcher, 2013). It is also a source of wood for pulp, furnishings, particleboard, and bioenergy. Despite its socio-economic and environmental roles, bamboo's stand is rapidly declining (Embaye, 2003a, Embaye, 2003b). The appropriate climatic conditions of a particular species, which could lead to movements from its original geological range, will be enhanced, decreased, or moved by environmental change (Gebrewahid et al., 2020).

Climate change is posing a threat to major plant biodiversity. Due to climate change, the effects of greenhouse gas emissions are projected to increase, causing irreversible impacts on ecosystems (van Vuuren et al., 2011; IPCC, 2014, 2019). In Africa, climate change is posing a threat and making many of the ecosystems and livelihoods vulnerable to climate-related risks (Conway and Schipper, 2011; IPCC, 2019). For example, a significant shift in the niche of the native species and habitat contraction due to climate change effects have been documented (Scholze et al., 2006; Mekasha et al., 2013; Tshwene-Mauchaza and Aguirre-Gutiérrez, 2019; Birhane et al., 2020). Moreover, climate change and human activities are among the factors that are strongly linked to the extinction of plant species globally. In Africa, this trend is anticipated to increase and gets more severe in the future.

The intentional or accidental movement of species by humans to regions far removed from their natural ranges has increased dramatically in frequency and extent in recent decades as human movements have become more global and international trade has increased. These pressures threaten the natural distribution and ecology of *O. abyssinica*. The increasing risk of extinction is owed to prolonged and hard-to-predict variations in the climate, which cause unprecedented changes in the natural limits of environmental indicators like rainfall and temperature. Since bamboo has great potential for climate change mitigation and adaptation (Yuen et al., 2017; Terefe et al., 2019; Nfornkah et al., 2020; Jyoti et al., 2021) understanding and modeling of its distributions are essential for safeguarding vulnerable and threatened species like *O. abyssinica* from further ecological and environmental stresses. Therefore, modeling the current and future distribution of *O. abyssinica* under changing climate conditions is a key step in developing actions for the conservation and management of the species (Qi et al., 2017; Gebrewahid et al., 2020) Furthermore, in Ethiopia, Cameron and Ghana for example, bamboo has been integrated into the national development plans and strategies (Minale and Abebe, 2020; Barnabas et al., 2020; Oduro et al., 2020); including green growth strategies and climate change plans (Minale and Abebe, 2020), and similarly, in Uganda, the bamboo industry is considered key for improving livelihoods in the dry area (Kalanzi et al., 2018; Mbazzira et al., 2018). Thus, this paper aimed to predict the current and future distribution of *O. abyssinica* in Africa in the face of climate change.

## 2 Materials and methods

### 2.1 Species description

*O. abyssinica* is one of the bamboo species commonly found in Africa, classified under the family Poaceae (Gramineae) (Embaye, 2003a, Embaye, 2003b; Fanshawe, 1972; Mulatu and Kindu, 2010). The species mainly grows in dense clumps of dry rocky hillsides in semi-humid lowland–savanna woodlands, along rivers, at altitudes ranging from 500 to 1800 m (Mulatu et al., 2016; Mulatu and Kindu, 2010) and annual rainfall of 700–1000 mm (Fanshawe, 1972; Sileshi and Nath, 2017; UNIDO, 2009). The species adapts well to poor soils and acts as a buffer in desert climate conditions (UNIDO, 2009). Currently, *O. abyssinica* grows in several East and West African regions, from Senegal to Ethiopia, Zimbabwe, Malawi, Mozambique, and Zambia (Embaye, 2000, 2003; Fanshawe, 1972; Hedberg et al., 2009; UNIDO, 2009)*. O. abyssinica* flowers every 7 years (Bekele-Tesemma and Tengnäs, 2007). In its native range, the population is presently declining due to its nature of gregarious flowering (INBAR, 2010), and management problems and the expansion of agricultural land (Embaye, 2000, 2003).

### 2.2 Species occurrence records

**Figure 1:**
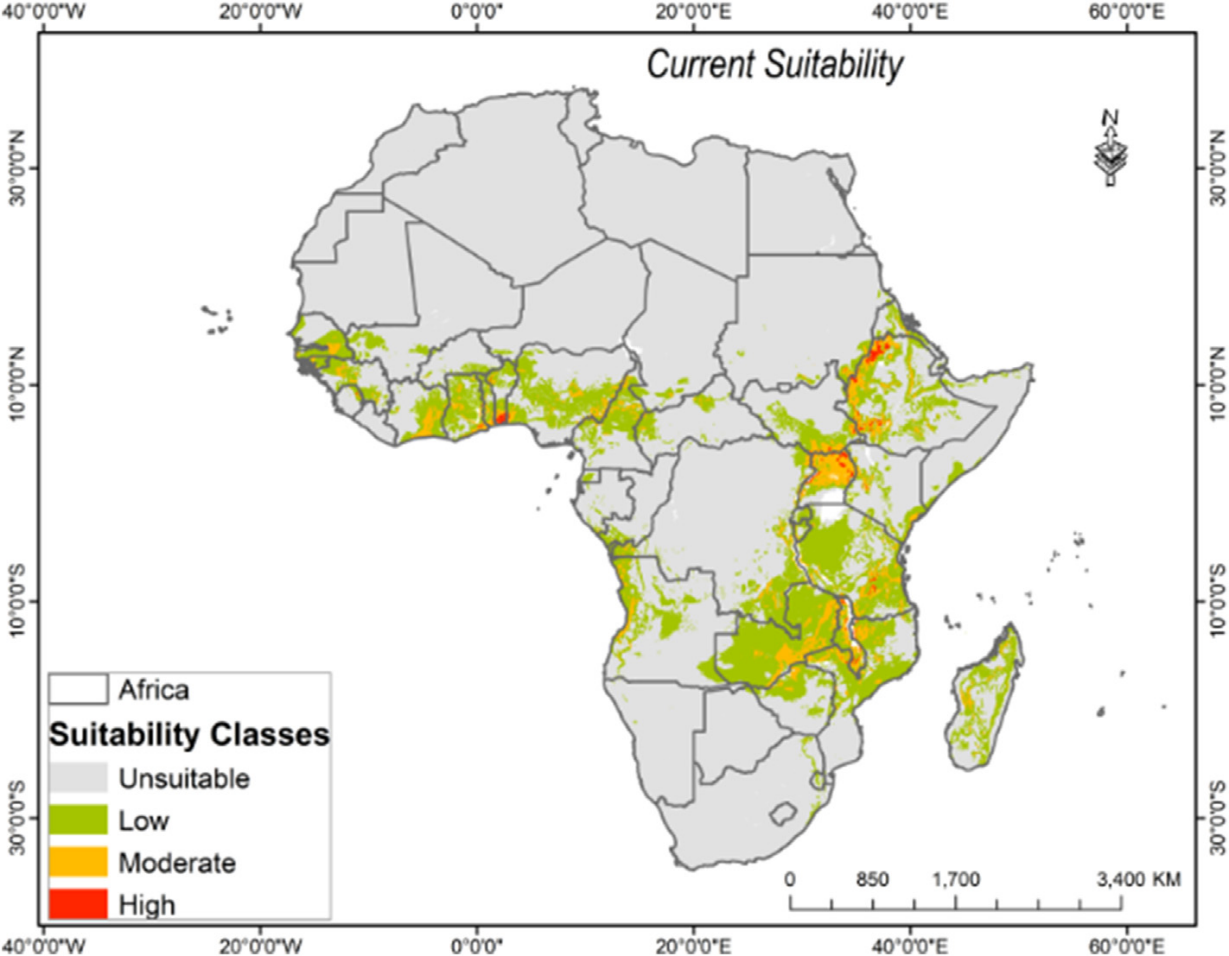
Habitat suitability range for *O. abyssinica* under current climatic condition.

Data on species presence was obtained from the Global Biodiversity Information Facility (GBIF: 290, https://www.gbif.org/), Flora of Zimbabwe (22:https://www.zimbabweflora.co.zw), Vegetationmap4africa (296: https://vegetationmap4africa.org/), databases, and GPS points collected by the authors (127) between 2011 and 2012 (Figure 1). After downloading the data, it was subjected to a quality examination and cleaning to ensure that there were no duplicate records. The gathered georeferenced points were visually inspected using ESRI ArcMap software, and duplicate entries were eliminated from the dataset. To that end, a total of seven hundred and thirty-five (735) spatially georeferenced *O. abyssinica* were collected from the current study sites. To reduce the influence of false absence, 2000 randomly distributed pseudo-absence points were generated over the geographical surface as suggested in (Barbet-Massin et al., 2012; Senay et al., 2013), and points closer than 5 km to the species' presence point were removed from the data based on (Eckert et al., 2020).

### 2.3 Climatic and environmental data

To estimate the existing and future distribution of the species, nineteen (19) bioclimatic variables were collected from the freely accessible WorldClim database version 2.1 (Fick and Hijmans, 2017: www.worldclim.org). The data is interpolated from more than 10,000 weather stations throughout the world and has a resolution of 5 arc minutes (Hijmans et al., 2005). The data was first obtained in GeoTiff (.tif) format at a global scale using the R getData raster package (Naimi, 2018:https://cran.r-project.org) in R, and then downscaled to the boundary of Africa using the clip tool in QGIS version 2.8. The bioclimatic data might have a collinearity issue, resulting in model instability and leading to the wrong interpretation of the model (Dormann et al., 2013). As a result, variance inflation factors (VIF) were utilized to identify the collinearity concerns within the bioclimatic variables (Marquardt, 1970). Environmental variables were subjected to pairwise Pearson's correlation coefficients in order to choose the variables with the highest correlation value (r > 0.7) and, as a result, those variables with higher correlation above the given threshold were eliminated from the model (Dormann et al., 2013). The most important environmental and climatic data selection done in this study was based on previously published studies in the literature (Gebrewahid et al., 2020), biologically relevant for the species survival, and statistically important in predicting the presence data for the selected species. Additionally, the vifstep function in the sdm R package was used to do this. A vifstep is a stepwise technique for removing variables that have a high level of linear correlation (i.e., larger than the threshold) with the other variables in the model (Naimi et al., 2014). Finally, during the modeling approach, a total of eight (8) bioclimatic variables (temperature and precipitation variables) were kept to find the ones that had the greatest influence on the distribution of *O. abyssinica*. Such as bioclimatic factors which are statistically important in predicting presence data for selected species, biologically relevant for survival of the species and collinearity.

The future effect of climate change on *O. abyssinica* distribution was projected based on the General Circulation Model (GCM) from the coupled climate model HadGEM2-AO for the Coupled Model Inter-comparison Project phase 5 (CMIP5). The Worldclim database (https://worldclim.org/data/v1.4/cmip5.html)was used to obtain the input data (Fick and Hijmans, 2017). This climate projection model (HadGEM2-AO) is selected because it is widely applied in species distribution modelling, and provides good coverage for the African continent. The RCPs scenarios were created to show climate scenarios with radioactive forcing anticipated to rise by 2.6, 4.5, 6, and 8.5 W per square meter (W/m-2) in the year 2100, according to van Vuuren et al. (2011), as well as widely used climate change modeling methods (IPCC, 2014). According to the report, under RCP 2.6, global carbon dioxide (CO2) emissions are expected to drop and reach zero by 2100. Furthermore, the global surface temperature is expected to rise by less than 2 degrees Celsius under this scenario. The RCP 6 is a set of scenarios that predict a drop in emissions after reaching a peak by 2080. Furthermore, in the RCP4.5 and RCP8.5 scenarios, atmospheric CO2 concentrations are expected to rise by 524.3 ppm (mg/L) and 677.1 ppm (mg/L), respectively, in the year 2070. Therefore, in this study to examine the habitat suitability range of *O. abyssinica* in the year 2050 (2041–2060) and the 2070s (2061–2080), RCP4.5 (the intermediate scenario for GHG emissions), RCP8.5 (the highest scenario for GHG emissions), and RCP2.6 (the minimum scenario for GHG emissions), were used (van Vuuren et al., 2011; IPCC, 2014, 2019).

### 2.4 Species distribution model

Species distribution models (SDMs) are the most powerful tools in the disciplines such as climate change impact assessment, ecological study, and conservation planning (Naimi, 2015), and in phylogeography study (Alvarado-Serrano and Knowles, 2014). Using presence and absence data, SDMs have a great ability to forecast species probability of occurrence in geographical areas (Srivastava et al., 2019). The SDM model can be run using the R package usdm, as well as the ensemble technique, which combines many statistical and machine learning algorithms (Marmion et al., 2009; Naimi and Araújo, 2016). The study used a total of five (5) SDM models including Multivariate Adaptive Regression Splines (MARS; regression-based), Boosted Regression Trees (BRT; boosting), Multilayer Perceptron Network (MLP; neural network), Random Forest (RF; machine learning-based), and Support Vector Machine (SVM). The selected model types are among the most prevailing models in the presence and absence of data types in species habitat suitability prediction (Naimi and Ara, 2016).

To estimate the current and future suitability maps of *O. abyssinica*, an ensemble modeling approach was adopted, which assimilates the different model results. This modeling approach has been recommended as one of the best in species distribution modeling (Araújo and New, 2007; Marmion et al., 2009; Gómez et al., 2018; Hao et al., 2020); and it reduces model uncertainty when using a single algorithm (Buisson et al., 2010; Alfaro et al., 2018; Turner et al., 2019). The ensemble approach is also reported as the most outperformed and consistent model in predicting different species by Stohlgren et al. (2010) and *Prosopis spp*. by Ng et al. (2018). However, a proper selection of parameters is required to reduce the possible model uncertainties.

### 2.5 SDM model performance evaluation

The receiver operating characteristics (ROC) based on the area under the curve (AUC) (Fielding & Bell, 1997) and true skills statistics (TSS) (Allouche et al., 2006) measure of metric statistics were used to assess the model's performance in predicting current and future suitability for *O. abyssinica*. The TSS values ranges from -1 to 1, while AUC values range from 0 to 1. The AUC and TSS range model categorization index were classified as reference (Thuiller et al., 2009). The indicator elucidates the various levels of model forecasting capability, ranging from low/fail to excellent. The default configuration of the model was set to 70% for training and 30% for evaluating the accuracy of the employed models (Araújo et al., 2005). For evaluation, we made 10 runs from a 4-fold cross-validation. We used a 4-fold cross-validation to create 10 runs for evaluation.

### 2.6 The current and future suitable area for *Oxytenanthera abyssinica*

The current and projected habitat suitability change analysis of *O. abyssinica* was conducted using the four Representation Concentration Pathways (RCPs), 2.6, 6., 4.5, and 8.5, for the years 2020 (current circumstances), 2050 (average for 2041–2060), and 2070 (average for 2061–2080) (van Vuuren et al., 2011). Based on the final distribution map developed from each climate scenario, four suitability groups were identified: not suitable (0–0.25), low suitable (0.25–0.5), moderately suitable (0.5–0.75), and extremely suitable (0.75–1) (Hamid et al., 2019; Weldemariam and Dejene, 2021). Then, to demonstrate the existing and future distribution of *O. abyssinica* in the research area, nine (9) maps were created, consisting of one map for current suitability (2020) and eight suitability maps connected with the four RCPs for the years 2050s and 2070s. The final ensemble maps were created using a weighted averaging method. Finally, for each range of suitability class, the area percentage was calculated in QGIS Version 2.8.

### 2.7 *Oxytenanthera abyssinica* habitat suitability change assessment

The ecological change over time was analyzed to investigate the likely influence of climate change on *abyssinica* suitable habitat ranges. As a result, four evaluation criteria were used to estimate current and future suitable habitat change based on (Dai et al., 2019; and Yan et al., 2020). The criteria were as follows: (i) unsuitable habitats: the areas where current and future (2050 and 2070) remains unsuitable habitats overlap; (ii) new suitable habitats: these areas that are currently unsuitable habitats but predicted to be converted into suitable habitats by the 2050 and 2070; (iii) suitable habitats that have not changed: these areas currently predicted as suitable habitats that overlap with future (2050 and 2070) suitable habitats; (iv) vulnerable areas: the areas currently suitable habitat which is projected as unsuitable habitat by the 2050s and 2070s.

To compute the effect of climate changes on the likelihood change rate of suitable habitat of *O. abyssinica* establishment under the current and future climatic scenarios indicators such as: suitable habitat expansion change rate in percentage (AC) was done following the method of (Sintayehu et al., 2021) and (Weldemariam and Dejene, 2021) based on;

(1) $$AC\;=\frac{\left(Af\;–\;Ac\right)}{Ac}*100$$

Where A_c_ is the current suitable habitat area predicted; A_f_ is the area predicted as suitable habitat by 2050 and 2070 climatic conditions; and A_cf_ is the suitable habitat found/overlapping in both the current and future climatic conditions (2050 and 2070).

## 3 Results

### 3.1 Evaluations of the model and its relative importance to variables

The mean AUC and TSS values for predicting the distribution of *O. abyssinica* were 93% and 74%, respectively (Table1). The RF algorithm performed better than all other models, whereas MARS was found to be the lowest performing algorithm.

**Table 1:** Mean performance statistics of the eight models using test datasets for predicting the current and future area suitability of *O. abyssinica* plant species under different climatic scenarios.

Measure of statistics	*Models*					
	SVM	RF	BRT	MARS	MLP	Mean value
**AUC**	0.92	0.98	0.91	0.90	0.94	0.93
**TSS**	0.71	0.86	0.67	0.66	0.79	0.74

Predictor’s variables affecting the distribution of *O. abyssinica* are presented in Table 2. The coldest quarter precipitation (boi19) was identified as the most important environmental predictor governing the distribution of *O. abyssinica* in Africa, followed by the coldest quarter mean temperature (bio11) and the warmest quarter precipitation (bio18), which explained the distribution of the species by 34.1%, 25.2%, and 23.7%, respectively (Table 2). On the contrary, precipitation during the driest period (bio14), mean temperature of the wettest quarter (bio8), and mean temperature during the warmest quarter (bio10) were the least influential environmental variables in determining the distribution of *O. abyssinica*, with an overall influence of 5.4%, 9.2%, and 10%, respectively. Overall, temperature-related factors (bio11 and bio3) and precipitation-related variables (bio18 and bio19) together contributed roughly 99.7% to the ensemble model prediction of *O. abyssinica* distribution. Similarly, topographic characteristics such as elevation and slope (31.6%) were shown to be among the most relevant factors affecting *O. abyssinica* distribution in Africa.

**Table 2:** The relative environmental variables contribution (%) to the ensemble model based on correlation metric.

Environmental variables	Description	Units	Percentage contribution
**Boi19**	Precipitation of coldest quarter	Mm	34.1%
**Bio11**	Mean temperature of coldest quarter	°C	25.2%
**Bio18**	Precipitation of warmest quarter	mm	23.7%
**Elevation**	Elevation	m	18.3
**Bio3**	Isothermality (Bio1/Bio7) × 100	°C	16.7%
**Slope**	Slope	Degree	13.3%
**Bio9**	Mean temperature of driest quarter	°C	13%
**Bio10**	Mean temperature of warmest quarter	°C	10%
**Bio8**	Mean temperature of wettest quarter	°C	9.2%
**Bio14**	Precipitation of driest period	mm	5.4%

### 3.2 Current and future potential distribution of *Oxytenanthera abyssinica* under climatic conditions

Under the current climatic conditions, the total suitable habitat (low to high suitability range) area for *O. abyssinica's* establishment was about 19.04% of the continent. About 15.46% of the area is categorized as low-suitable, 3.32% as moderate, and 0.26% as a highly suitable habitat for *O. abyssinica* establishment. However, in the current climatic scenario, about 80.97% of the continent remains potentially unsuitable for the species (Table 3 and Figure 1).

**Table 3:** Total suitability and suitable habitat change for *O. abyssinica* in percentage with respect to the current distribution.

Total suitability in (%)					
Years	Scenarios	Not suitable	Low	Moderate	High
Current		80.97	15.46	3.32	0.26
2050	RCP2.6	73.48	22.19	4.09	0.24
	RCP4.5	71.62	23.52	4.63	0.24
	RCP6.0	73.77	21.80	4.24	0.20
	RCP8.5	71.99	23.15	4.65	0.21
2070	RCP2.6	75.46	20.36	3.95	0.23
	RCP4.5	70.25	24.31	5.24	0.20
	RCP6.0	71.21	23.84	4.78	0.17
	RCP8.5	69.50	26.01	4.40	0.10

Most of the sub-Saharan African countries and the Sahel zone remain the main hotspot areas for *O. abyssinia*, under the current climatic conditions. Countries like the United Republic of Tanzania, Zambia, Mozambique, the Democratic Republic of the Congo (southeast), Madagascar, Ethiopia, Nigeria, South Sudan (southern), Cameroon, Central African Republic, Guinea, Ghana, Uganda, Malawi, Kenya, Eritrea, and Cote d'Ivoire were found among the most suitable areas for *O. abyssinica*. Similarly, our model predicted the presence of small suitable habitat patches for *O. abyssinica* in countries like Liberia, Cape Verde, Lesotho, Guinea-Bissau, Niger, Gambia, and Comoros (Figure 1).

**Figure 2:**
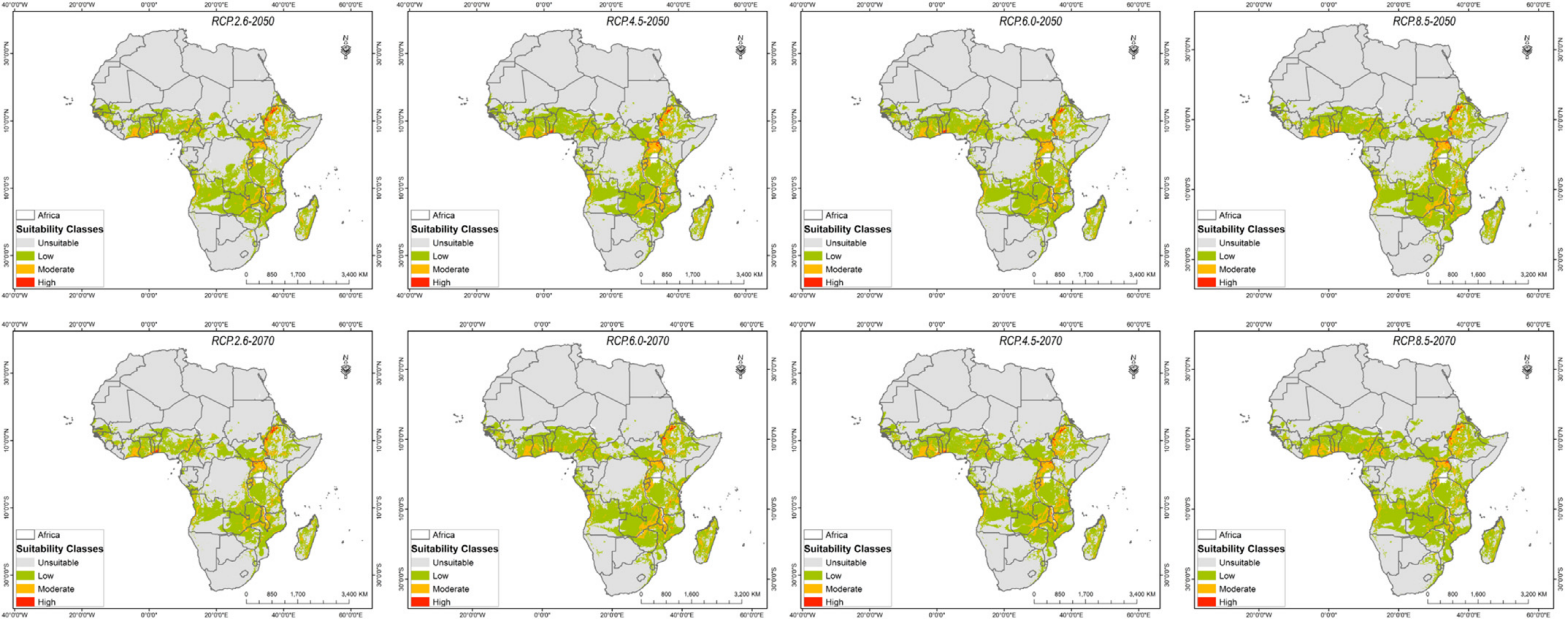
*O. abyssinica* suitability classes under future projected climatic condition for different RCPs (RCP2.6, RCP4.5, RCP6.0, and RCP8.5) to the year 2050 and 2070.

Figure 2 shows the potential of suitable habitat for *O. abyssinica* under RCP2.6, 4.5, 6, and 8.5 scenarios for the 2050s and 2070s years. The model predicted an increasing trend in suitable habitat for the low and moderate suitability classes in these periods, whereas a progressive decrease in suitability was predicted for the highly suitable areas (Table 4; Figure 2). By the mid-century (the 2050s), areas which were predicted as highly suitable under RCP2.6, RCP4.5, RCP6, and RCP8.5 climatic scenarios were 0.24%, 0.24%, 0.20%, and 0.21%, respectively. For the same period, the moderate suitable area for the RCP2.6, RCP4.5, RCP6, and RCP8.5 climatic scenarios was 4.09%, 4.63%, 4.24%, and 4.65%, respectively. Furthermore, the ensemble prediction by the end of the century (the 2070s) of the moderately suitable areas under RCP 2.6, RCP 4.5, RCP 6, and RCP 8.5 climatic scenarios in their order was found to be 3.95%, 5.24%, 4.78%, and 4.4% of the continent. During the same period (the 2070s), the highly suitable habitat was projected as 0.23% in RCP2.6, 0.2% in RCP4.5, 0.17% in RCP6, and 0.1% in RCP8.5 climatic scenarios. Our study established that for all RCP's except RCP2.6 in both periods (the 2050s and 2070s), the total area predicted under the low suitability class range has shown a progressive increase over the continent (Table 4 and Figure 2). Similarly, the model prediction has shown a consistent decrease in the suitable area for *O. abyssinica* under the moderate suitable classes for the RCP2.6 and 8.5 to the projected years of 2050 and 2070.

**Table 4:** Suitable habitat change rate of *O. abyssinica* in Africa under different climatic scenarios (RCP) for the year 2050 and 2070s.

	Suitable habitat change rate (%)-AC				
*Year*	*RCPs*	*Not suitable*	Low	Moderate	High
2050	RCP2.6	-9.25	43.54	23.29	-6.74
	RCP4.5	-11.55	52.16	39.37	-7.46
	RCP6.0	-8.89	41.00	27.63	-21.11
	RCP8.5	-11.09	49.79	40.01	-19.17
2070	RCP2.6	-6.80	31.71	18.83	-9.99
	RCP4.5	-13.24	57.28	57.87	-23.31
	RCP6.0	-12.05	54.26	43.95	-34.76
	RCP8.5	-14.16	68.25	32.47	-62.49

### 3.3 Suitability change for *Oxytenanthera abyssinica* to climatic conditions

**Figure 3:**
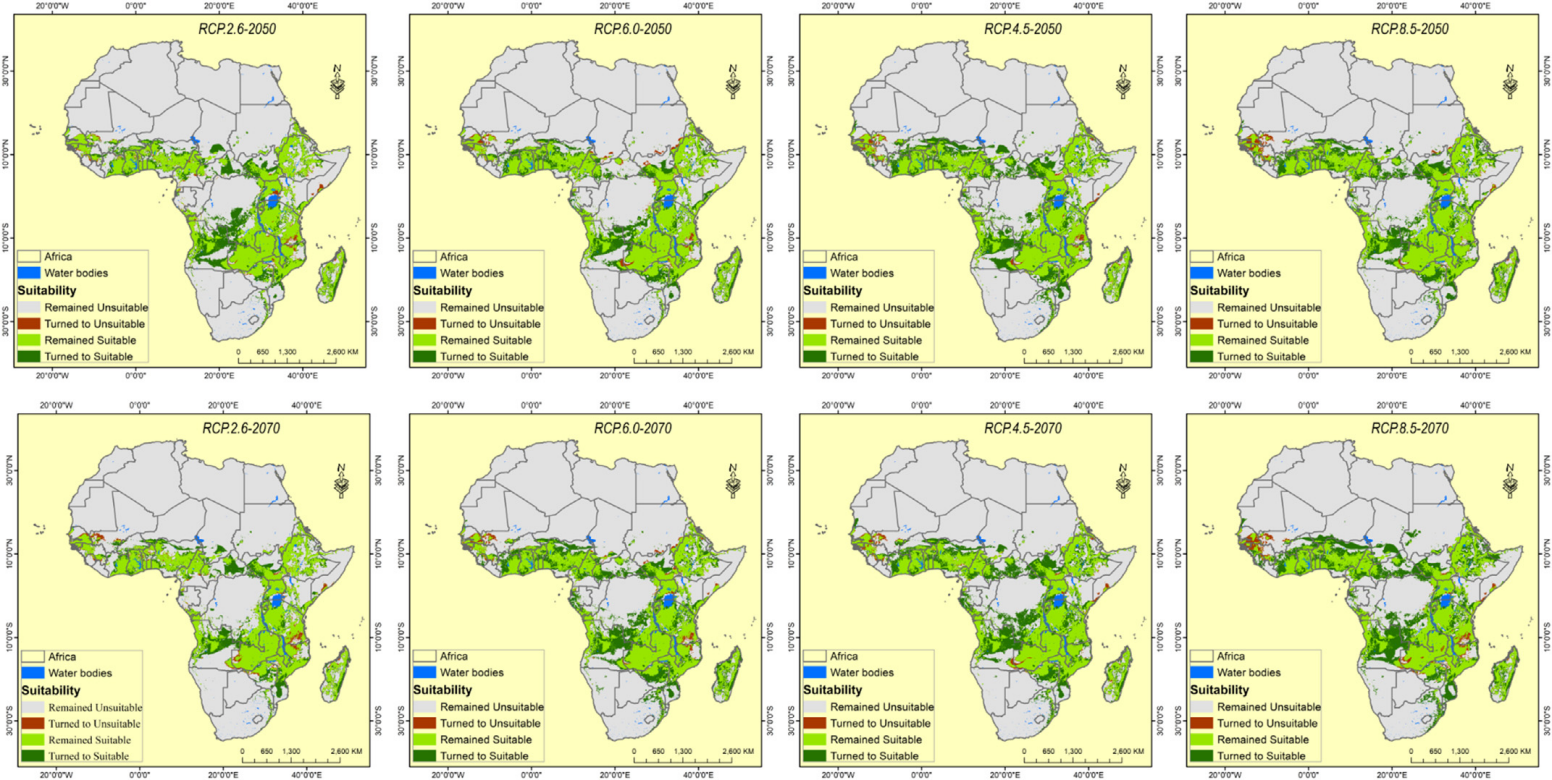
Changing in suitable habitat of *O. abyssinica* distribution from current climatic conditions to future climatic conditions in the 2050 and 2070 for RCP2.6, RCP4.5, RCP6.0, and RCP8.5.

Compared to the current climatic conditions, the future habitat suitability for *O. abyssinica* establishment is anticipated to increase across the continent in the mid (2050) and end (2070) century, except for the RCP2.6 and RCP8.5 (moderate suitability class range). Nevertheless, the highly suitable area was projected to decrease in all RCP's for both periods (2050s and 2070s). Correspondingly, the future projection has shown a decreasing trend in the non-suitable areas in all considered RCP's. Overall, our habitat suitability assessment demonstrated a significant impact of future climate change on the potential establishment of *O. abyssinica* in Africa (Table 4 and Figure 3).

## 4 Discussion

The consensus model has a respectable range of mean AUC (93%) and TSS (74%) values, putting it quite close to the optimal prediction range. Furthermore, the visual evaluation of the sample sites using overlay analysis proved this. The result demonstrated the robustness of ensemble models in predicting the targeted species and is consistent with previous research (Swets, 1988; Marmion et al., 2009; Thuiller et al., 2009). Our study revealed precipitation during the cold and warmer quarters, temperature during the cold quarter, and isothermality as the main climatic factors affecting the distribution of *O. abyssinica* in the study area. Additionally, topographic factors such as elevation and slope have shown a considerable impact on the distribution of the species.

Climate change appears to be the most significant environmental factor influencing the range of *O. abyssinica* distribution. The population distribution of *O. abyssinica* in its native habitat is under threat as a result of multiple interrelated human-induced and natural processes (Embaye, 2003a, Embaye, 2003b), with climate change being the most significant of these. Our model predicted that *O. abyssinica* plant species would have a more restricted geographic range and thus have a lower occurrence probability with future climate warming; typically, a large decrease in the species' distribution area was observed under the high-intensity climatic projection compared to the low-intensity climatic scenarios. Previous research findings has clearly proven the impact of long-term climate change on native plant species shifting from their original niche ranges to new locations (Eeley et al., 1999; Qin et al., 2017). Our findings also verified the relevance of precipitation-related environmental variables, which are posing a serious threat to the existence of many species, including *O. abyssinica*. Gebrewahid et al. (2020); and Hawinkel et al. (2016) reported that environmental variables such as precipitation are the most significant factors affecting the distribution of *O. abyssinica*. Upson et al. (2016) and Sintayehu (2018) revealed the potential effect of extreme climate warming on modifying plant growth and the increasing vulnerability to climate change impact within its native range. In the long run, the cumulative impact of all climate extreme events will also accelerate the total alteration of the ecosystem and its structure (Zhang et al., 2022). Furthermore, climate change and its associated warming might also hamper the rate of seed germination and regeneration of the plants, subsequently limiting their habitat range and abundance (Mondoni et al., 2012), which is typically the case in the African climate. Moreover, Zhong et al. (2010) and Wu et al. (2014) establish a strong correlation between plant richness and seasonal climate variation, with plant distribution directly linked to precipitation and temperature.

Designing a proper management and conservation strategy which takes into account the existing dynamics will help *O. abyssinica* to establish well in its current area (Abebe et al., 2021), where it otherwise may lose its habitat as a result of such unanticipated pressures. Thus, a collective approach involving the implementation of socio-ecological frameworks for landscape planning and conservation that links human beings with their socio-cultural and ecological mechanisms will be valuable for the sustainable utilization and survival of the species in its current habitat range (Abebe et al., 2021; Gebrewahid et al., 2020; Lamsal et al., 2017; Eeley et al., 1999). Above all the fast growing nature of the bamboo plant combined with its adaptive capability on degraded and marginal land has made this plant species to receive huge attention by ecologist in eco-restoration of degraded land (Mishra et al., 2014; Donfack, 2020), carbon stock and sequestration (Abebe et al., 2021) and for future climate change adaptation and mitigation (Lobovikov et al., 2012). This suggests the need for comprehensive and integrated biodiversity conservation and restoration programs aimed at increasing the highly suitable habitat for *O. abyssinica* in its original habitats.

The SDM model in this study only took into account the most important bioclimatic and topographic factors; however, land-cover, vegetation index, including other anthropogenic and natural factors that might threaten the species survival, are not integrated in the model, though they are important variables in improving the model results. As a result, we strongly suggest future studies take these variables into account. Nevertheless, the absence of future data could limit their use for studies. Despite the many expectations and uncertainties in species distribution models, yet SDM remain an important data source for projecting species distributions and appraising scientific adaptation strategies for mitigating the effect of future warming on plant species at various scales (Ackerly et al., 2010; Yates et al., 2010).

## 5 Conclusion

Despite a steady shrinkage in the highly suitable area, the current findings show that *O. abyssinica* distributions across the continent are considered good. Areas regarded as low and moderately suitable for *O. abyssinica* under the intermediate and end-of-century climatic scenarios for the 2050s and 2070s are anticipated to increase across the study area. However, our current study showed a significant contraction of the highly suitable area range for *O. abyssinica* establishment across the continent. If the current trend continues, a total habitat shift from its natural niche will be anticipated shortly. Therefore, this study indicates the need to incorporate future climate change scenarios into the existing conservation and management strategies and seeks possible recommendations for protecting the species in its current niche. Finally, this research enabled us to understand the most important bioclimatic factors affecting the distribution of *O. abyssinica* on the continent of Africa.

## Declarations

### Author contribution statement

Weldemariam Ch. Elias: Conceived and designed the experiments; Performed the experiments; Contributed materials, analysis tools or data; Analyzed and interpreted the data; Wrote the paper.

Dejene W. Sintayehu & Bobasa F. Arbo: Conceived and designed the experiments; Analyzed and interpreted the data; Wrote the paper.

Abraha K. Hadera: Analyzed and interpreted the data.

### Funding statement

This research did not receive any specific grant from funding agencies in the public, commercial, or not-for-profit sectors.

### Data availability statement

Data will be made available on request.

### Declaration of interest’s statement

The authors declare no conflict of interest.

### Additional information

No additional information is available for this paper.
